# Clinical Utility and Diagnostic Accuracy of ROMA, RMI, ADNEX, HE4, and CA125 in the Prediction of Malignancy in Adnexal Masses

**DOI:** 10.3390/cancers16223790

**Published:** 2024-11-11

**Authors:** Giulia Spagnol, Matteo Marchetti, Massimo Carollo, Sofia Bigardi, Marta Tripepi, Emma Facchetti, Orazio De Tommasi, Amerigo Vitagliano, Francesco Cavallin, Roberto Tozzi, Carlo Saccardi, Marco Noventa

**Affiliations:** 1 Unit of Gynecology and Obstetrics, Department of Women and Children’s Health, University of Padua, 35122 Padua, Italy; 2 Department of Diagnostics and Public Health, University of Verona, 37129 Verona, Italy; 3 Department of Primary Care, ULSS 1 Dolomiti, 32100 Belluno, Italy; 4 1st Unit of Obstetrics and Gynecology, Department of Biomedical and Human Oncological Science (DIMO), University of Bari, Policlinico, 70121 Bari, Italy; 5 Independent Statistician, 36020 Solagna, Italy

**Keywords:** ultrasound, serum markers, IOTA, ADNEX, ovarian cancer

## Abstract

Objective: We aimed to compare the clinical utility and diagnostic accuracy of the ADNEX model, ROMA score, RMI I, and RMI IV, as well as two serum markers (CA125 and HE4) in preoperative discrimination between benign and malignant adnexal masses (AMs). Methods: We conducted a retrospective study extracting all consecutive patients with AMs seen at our Institution between January 2015 and December 2020. Accuracy metrics included sensitivity (SE), specificity (SP), and area under the receiver operating characteristic curve (AUC), and their 95% confidence intervals (CI) were calculated for basic discrimination between AMs. Model performance was evaluated in terms of discrimination ability and clinical utility (net benefit, NB). Results: A total of 581 women were included; 481 (82.8%) had a benign ovarian tumor and 100 (17.2%) had a malignant tumor. The SE and SP of CA125, HE4, ROMA score, RMI I, RMI IV, and ADNEX model were 0.60 (0.54–0.66) and 0.80 (0.76–0.83); 0.39 (0.30–0.49) and 0.96 (0.94–0.98); 0.59 (0.50–0.68) and 0.92 (0.88–0.95); 0.56 (0.46–0.65) and 0.98 (0.96–0.99); 0.54 (0.44–0.63) and 0.96 (0.94–0.98); 0.82 (0.73–0.88) and 0.91 (0.89–0.94), respectively. The overall AUC was 0.76 (0.74–0.79) for CA125, 0.81 (0.78–0.83) for HE4, 0.82 (0.80–0.85) for ROMA, 0.86 (0.84–0.88) for RMI I, 0.83 (0.81–0.86) for RMI IV, and 0.92 (0.90–0.94) for ADNEX. The NB for ADNEX was higher than other biomarkers and models across all decision thresholds between 5% and 50%. Conclusions: The ADNEX model showed a better discrimination ability and clinical utility when differentiating malignant from benign Ams, compared to CA125, HE4, ROMA score, RMI I, and RMI IV.

## Simple Summary

A transvaginal ultrasound examination is often used in clinical practice as the standard first-line imaging investigation for the assessment of adnexal masses. The accurate identification of malignancy ensures timely intervention and allows for the referral of the patient to a specialized center, particularly given the aggressive nature of ovarian cancer. Over the years, various serum biomarkers (CA125, HE4) and ultrasound-based models (e.g., Risk of Malignancy Algorithm (ROMA score), Risk of Malignancy Index (RMI I-IV), and Assessment of Different NEoplasia in the adneXa (ADNEX model) have been proposed to predict the malignancy risk of an adnexal mass. Comparing these models is essential to determine which one offers the best diagnostic accuracy and clinical utility.

## 1. Introduction

Ovarian cancer is the most lethal of all gynecologic malignancies, with approximately 60% of cases diagnosed at an advanced stage, leading to a poor long-term prognosis and high mortality rates [1,2]. Adnexal masses (AMs) are commonly detected during gynecological examinations, and while the majority of these masses are benign, distinguishing between benign and malignant lesions is critically important for determining the appropriate course of management [3,4]. Accurate identification of malignancy ensures timely intervention, particularly given the aggressive nature of ovarian cancer.

Transvaginal ultrasound (TV-US) remains the primary imaging modality for evaluating adnexal masses. Subjective assessment by an experienced examiner is regarded as the most effective method for differentiating between benign and malignant AMs, given an examiner’s ability to capture subtle features that may suggest malignancy [5,6]. However, to reduce diagnostic uncertainty, several predictive models have been developed in recent years, aimed at improving the identification of patients at high risk for ovarian cancer [7,8]. Among these, the ovarian cancer-associated tumor marker CA125 has long been used to detect malignancy in women with pelvic masses. However, its diagnostic accuracy is notably limited, particularly in detecting early-stage ovarian cancer, where sensitivity and specificity may be insufficient for reliable diagnosis [9,10]. Another biomarker, Human Epididymis Protein 4 (HE4), has demonstrated similar performance to CA125 and is often utilized alongside it [11,12]. The combination of these two markers forms the basis of the Risk of Malignancy Algorithm (ROMA), which takes into account serum CA125 and HE4 levels, as well as menopausal status, to estimate malignancy risk [13,14]. In 1990, Jacobs et al. introduced the Risk of Malignancy Index (RMI), an algorithm that incorporates ultrasound findings, CA125 levels, and menopausal status to predict the likelihood of malignancy in adnexal masses. The RMI has since become a widely used tool in clinical practice, with multiple studies showing comparable diagnostic accuracy between the RMI and the ROMA model, particularly in postmenopausal women [15,16].

In 2014, the International Ovarian Tumor Analysis (IOTA) group developed a more advanced predictive model, the Assessment of Different NEoplasia in the adneXa (ADNEX). This model integrates three clinical variables and six ultrasound features to predict the likelihood of different types of ovarian neoplasms, including benign ovarian tumors, borderline ovarian tumors (BOT), stage I epithelial ovarian cancer, stages II–IV epithelial ovarian cancer, and ovarian metastasis [17,18,19,20].

The aim of this study was to evaluate and compare the diagnostic performances, in terms of discrimination ability, calibration, and net benefit (clinical utility), of the biomarkers (i.e., CA125 and HE4) and four models ROMA, RMI I, RMI IV, and ADNEX, in preoperative discrimination between benign and malignant AMs in a gynecological tertiary cancer center in northern Italy.

## 2. Materials and Methods

### 2.1. Study Design

This is a retrospective study based on a prospectively maintained database of consecutive patients, aimed at the preoperative differentiation of benign and malignant AMs. We report the study in accordance with the Transparent Reporting of a multivariable prediction model for Individual Prognosis or Diagnosis (TRIPOD guidelines) [21]. All patients gave written informed consent for the anonymous use of their data according to European privacy law. This study was approved by the institutional review board (IRB: 42n/AO/2020).

### 2.2. Data Source and Study Cohort

Anonymized data were collected using electronic health records. We included all consecutive patients with diagnosis of AMs who underwent surgery at the Department of Gynaecology and Obstetrics of the University of Padova between January 2015 and December 2020. Additional inclusion criteria were as follows: (i) patients who underwent TV-US within a maximum of three months prior to the surgical procedure; (ii) CA125 and HE4 levels measured within three months prior to surgery. Exclusion criteria were as follows: (i) age less than 18 years; (ii) ongoing pregnancy; (iii) patients with a previously confirmed diagnosis of malignancy; (iv) no consent for the use of data. TV-US was the primary approach; also, we performed transabdominal ultrasounds when the lesion was too large. TV-US was performed by an experienced gynecologist or by a gynecologist in training under supervision. The ultrasound machines used were Voluson E8 and S10 (GE Healthcare, Zipf, Austria), with 5.0–9.0 MHz TV probes and 1.0–5.0 MHz transabdominal probes. All women received specific surgical treatment at our clinic based on their age and the clinical diagnosis. All specimens were examined by an experienced gynecologic pathologist for histology and grade, then staged (I–IV) according to the International Federation of Gynecology and Obstetrics (FIGO) classification in case of malignancy [22]. For each patient we collected the following data: (i) age at surgery, (ii) menopausal status: premenopausal and postmenopausal, (iii) pathology features, (iv) CA125 and HE4 serum concentrations, (v) the results of four different models for the evaluation of AMs, in particular ROMA score, RMI I, RMI IV, and ADNEX.

### 2.3. Biomarkers and Prediction Models

The study assessed two serum biomarkers (CA125 and HE4) and four predictive models for the pre-operative evaluation of AMs: ROMA score, RMI I, RMI IV, and ADNEX. The investigator who calculated the score of the diagnostic models was blinded to surgery and pathology reports. CA125 and HE4 were measured with electrochemiluminescence immunoassay (ECLIA) within three months prior to surgery. The established cut-off levels used were CA125 > 35 U/mL and HE4 > 70 pmol/L in premenopausal and >140 pmol/L in post-menopausal women, respectively.

ROMA estimates the risk of malignancy based on menopausal status, HE4, and CA125 serum levels [13]. Two different formulas were used for premenopausal and postmenopausal patients as appropriate; the performance of ROMA was analyzed for all patients combined. According to the manufacturer’s instructions, a ROMA score of ≥11.4% for Pre-M and ≥29.9% for Post-M indicates a high risk of ovarian cancer [13,14]. RMI I combines the serum level of CA125, menopausal status, and ultrasound findings into a single score. A score greater than 200 suggests a high risk of malignancy [15]. RMI IV is a modified version of RMI I, designed to enhance its diagnostic accuracy in certain clinical settings, and was defined as RMI × S, where S is based on the size of the lesion (i.e., S = 1 if the maximum diameter of the lesion is <7 cm, S = 2 if it is ≥7 cm). An RMI IV score greater than 450 was used as the cut-off level for ovarian cancer [15]. ADNEX incorporates three clinical (i.e., age, type of center, and optionally, serum CA125) and six ultrasound features (the maximum tumor diameter, the proportion of solid tissue (defined as the ratio of the largest solid component’s diameter to the lesion’s maximum diameter), the presence of more than ten cyst locules, the number of papillary structures, the presence of acoustic shadows, and the presence of ascites) [17]. We used ADNEX with CA125. The commonly accepted malignancy risk threshold for ADNEX is 10% [17].

### 2.4. Reference Standard

The final histology of the surgical specimen served as the reference standard, with results categorized as benign, borderline, or malignant, based on the World Health Organization (WHO) guidelines, and malignancies were staged (I–IV) according to the FIGO classification [22,23]. The pathologists received clinical information, as per local practice. In our statistical analysis, borderline ovarian tumors (BOTs) were classified as malignant.

### 2.5. Statistical Analysis

The sample size was initially based on the rule of thumb of 100 observations in the smallest outcome category, as suggested by Steyerberg [24]. However, all patients meeting the inclusion criteria were included in the study to ensure comprehensive data collection and a post hoc power analysis was conducted to evaluate the adequacy of the sample size for detecting a statistically significant area under the receiver operating characteristic curves (AUCs), compared to a null hypothesis of AUC = 0.5, which represents no discriminative ability [25].

To evaluate the ability of the models and biomarkers to discriminate between benign and malignant AMs, we computed key performance metrics, including AUC, sensitivity (SE), specificity (SP), negative predictive values (NPVs), positive predictive values (PPVs), Youden’s index, and F_1_-score, across a range of decision thresholds to classify patients into low-risk (risk < threshold) or high-risk (risk ≥ threshold) categories. The specific thresholds for the calculated risk of malignancy were 1%, 3%, 5%, 10%, 15%, 20%, 25%, 30%, 40%, and 50%. DeLong and Wilson methods were used to calculate 95% confidence intervals (CIs) for AUCs and SE/SP, respectively. The calibration of risk estimates was assessed using a logistic recalibration model, generating a calibration plot and calculating both the intercept and slope of the calibration curves. These measures were used to evaluate the agreement between predicted probabilities and observed outcomes, indicating whether the models tended to overestimate or underestimate the actual risk of malignancy. We also calculated the net benefit (NB) for decision thresholds between 5% and 50% for the risk of malignancy, which was used to determine the clinical utility of each model in deciding which patients should be offered treatment for malignancy [26].

Additionally, we calculated performance metrics based on the recommended decision thresholds for all biomarkers and models (i.e., categorizing patients in a binary manner as either low-risk or high-risk). In a prespecified subgroup analysis, we calculated performance metrics for premenopausal and postmenopausal patients separately. Missing values were considered purely random missingness and, as such, were handled using multiple imputation (i.e., ten imputed datasets were generated). Descriptive statistics were used to summarize data. Continuous variables were reported as medians and interquartile ranges (IQR), while categorical variables were summarized as frequencies and percentages. All analyses were performed using R 4.4.1 (packages “amelia”, “caret”, “pROC”, “psych”, “rms”, and “rmda”) (R Foundation for Statistical Computing, Vienna, Austria) [27].

## 3. Results

### 3.1. Patient and Tumor Characteristics

Overall, a total of 581 patients were included. The median age (IQR) for the study group was 49 years (range 39–61 years). We collected data from 312 (53.7%) pre-menopausal women and 268 (46.3%) post-menopausal women. Pathology tests reported 62 (10.7%) ovarian cancers, 38 (6.5%) BOTs, and 481 (82.8%) benign tumors (Table 1). Among these patients, the most invasive tumors were HGSOC (44, 70.9%), eight were endometrioid (12.9%), and two were mucinous (3.2%); seven other types were also present (15.6%). Of them, 40 (64%) were diagnosed at FIGO stages III–IV.

### 3.2. Model Discrimination

**Figure 1:**
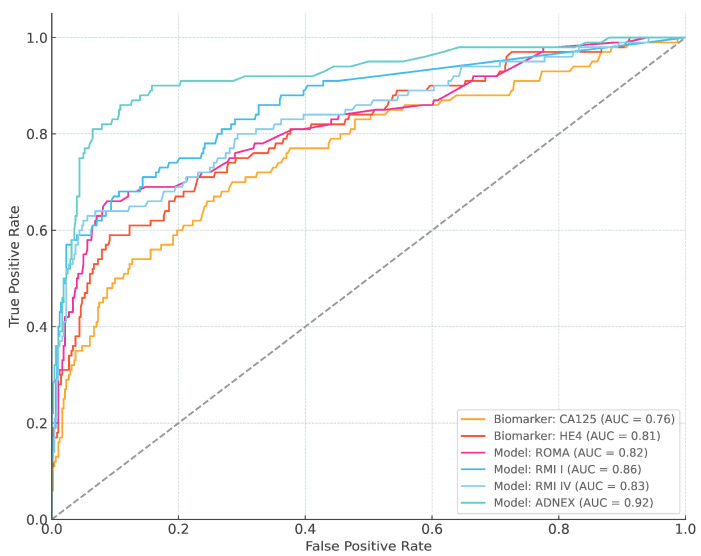
The area under the receiver operating characteristic curve (AUC) of CA125, HE4, ROMA, RMI I–IV, and ADNEX model.

The overall AUC (95% CI) was 0.76 (0.74–0.79) for CA125, 0.81 (0.78–0.83) for HE4, 0.82 (0.80–0.85) for ROMA, 0.86 (0.84–0.88) for RMI I, 0.83 (0.81–0.86) for RMI IV, and 0.92 (0.90–0.94) for ADNEX (Figure 1 and Table 2). The pairwise comparison among the different biomarkers and models is shown in Table S1; we found that ADNEX performed significantly better in terms of AUC when compared to RMI, IV, and ROMA score. There were no significant differences between RMI I, IV, and ROMA score.

At the selected threshold of predicted probabilities, according to the maximum Youden index, the best cutoffs were 29.8 kU/L and 50.2 kU/L for CA125 (thresholds of 30% and 50%, respectively, Youden index 0.41), 50.1 pmol/L for HE4 (threshold 50%, Youden index 0.46), 15% and 20% for ROMA (Youden index 0.55), 50.7 for RMI I (threshold 50%, Youden index 0.56), 50 for RMI IV (threshold 50%, Youden index 0.42), and 10% for ADNEX (Youden index 0.74) (Table 2).

In a post hoc analysis, evaluating data across the full range of values, the best cutoffs according to the maximum Youden index were 25.8 kU/L for CA125 (SE 0.70, SP 0.72, Youden index 0.4152), 69.3 pmol/L for HE4 (SE 0.59, SP 0.91, Youden index 0.4985), 18.7% for ROMA (SE 0.66, SP 0.91, Youden index 0.5727), 69.3 for RMI I (SE 0.68, SP 0.89, Youden index 0.5727), 254.4 for RMI IV (SE 0.63, SP 0.94, Youden index 0.5739), and 8.1% for ADNEX (SE 0.86, SP 0.89, Youden index 0.7519).

When used at the recommended decision thresholds, CA125 had a SE of 0.60 (0.54–0.66), HE4 of 0.39 (0.30–0.49), ROMA of 0.59 (0.50–0.68), RMI I of 0.56 (0.46–0.65), RMI IV of 0.54 (0.44–0.63), and ADNEX of 0.82 (0.73–0.88) (Table 3). SP was 0.80 (0.76–0.83) for CA125, 0.96 (0.94–0.98) for HE4, 0.92 (0.88–0.95) for ROMA, 0.98 (0.96–0.99) for RMI I, 0.96 (0.94–0.98) for RMI IV, and 0.91 (0.89–0.94) for ADNEX (Table 3).

### 3.3. Calibration and Net Benefit

**Figure 2:**
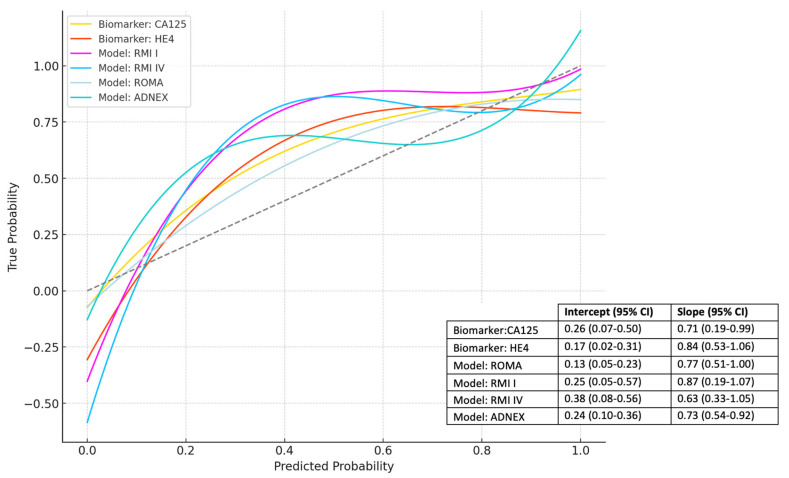
Overall calibration curves for the estimated risk of malignancy. Abbreviations: ADNEX = assessment of different neoplasia in the adnexa; CA125 = ovarian cancer-related tumor marker; CI = confidence interval; HE4 = human epididymis protein 4; RMI = risk of malignancy index; ROMA = risk of ovarian malignancy algorithm.

Calibration curves, along with the corresponding intercepts and slopes, are provided in Figure 2. The ROMA score had a calibration intercept of 0.13 (0.05–0.23) with a slope of 0.77 (0.51–1.00); the ADNEX model had a calibration intercept of 0.24 (0.10–0.36) with a slope of 0.73 (0.54–0.92); and RMI I and RMI IV had calibration intercepts of 0.25 (0.05–0.57) and 0.38 (0.08–0.56), respectively, with slopes of 0.87 (0.19–1.07) and 0.63 (0.33–1.05), respectively.

**Figure 3:**
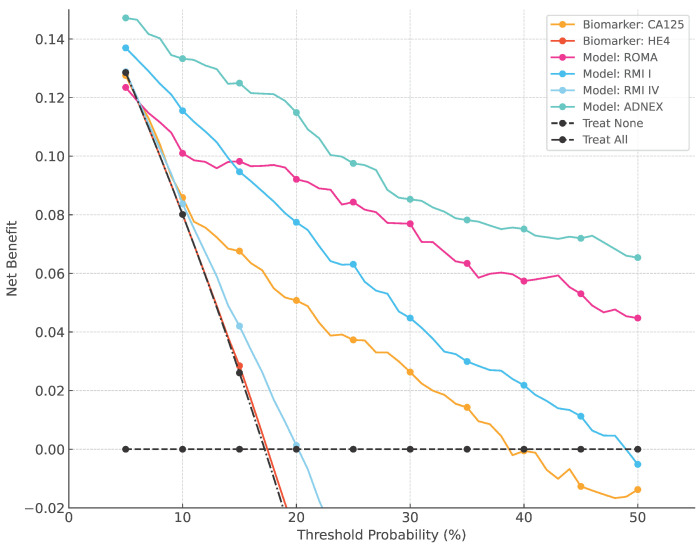
Overall decision curves. Higher net benefit implies a higher clinical utility, conditional on the decision threshold.

The NB for ADNEX was higher than that of other biomarkers and models across all decision thresholds between 5% and 50% (Figure 3). Additionally, the “treat all” strategy consistently showed lower NB compared to all tested biomarkers and models except HE4.

### 3.4. Subgroup Analysis

In the premenopausal patient subset, AUC (95% CI) was 0.67 (0.62–0.72) for CA125, 0.77 (0.72–0.81) for HE4, 0.77 (0.73–0.81) for ROMA, 0.81 (0.77–0.85) for RMI I, 0.77 (0.72–0.81) for RMI IV, and 0.90 (0.87–0.94) for ADNEX. Other performance metrics are shown in Table S2. Using recommended cutoffs, CA125 had a SE of 0.47 (0.40–0.54), HE4 of 0.39 (0.30–0.49), ROMA of 0.42 (0.35–0.50), RMI I of 0.37 (0.30–0.45), RMI IV of 0.37 (0.29–0.45), and ADNEX of 0.76 (0.72–0.80) (Table S3). SP was 0.72 (0.65–0.79) for CA125, 0.97 (0.95–0.99) for HE4, 0.92 (0.87–0.97) for ROMA, 1.00 (0.98–1.00) for RMI I, 0.98 (0.96–1.00) for RMI IV, and 0.94 (0.92–0.96) for ADNEX. Considering the postmenopausal patient subset, AUC (95% CI) was 0.84 (0.81–0.88) for CA125, 0.82 (0.79–0.85) for HE4, 0.87 (0.84–0.90) for ROMA, 0.89 (0.86–0.91) for RMI I, 0.88 (0.85–0.91) for RMI IV, and 0.93 (0.91–0.95) for ADNEX. Other performance metrics are shown in Table S4.

Using recommended cutoffs, CA125 had a SE of 0.68 (0.64–0.72), HE4 of 0.39 (0.30–0.48), ROMA of 0.69 (0.63–0.75), RMI I of 0.68 (0.64–0.72), RMI IV of 0.65 (0.61–0.69), and ADNEX of 0.85 (0.83–0.88) (Table S5). SP was 0.91 (0.87–0.94) for CA125, 0.95 (0.93–0.98) for HE4, 0.91 (0.88–0.94) for ROMA, 0.95 (0.93–0.97) for RMI I, 0.95 (0.92–0.97) for RMI IV, and 0.88 (0.85–0.91) for ADNEX.

## 4. Discussion

This study provides a comprehensive evaluation of the discrimination ability, calibration, and clinical utility of the RMI I–IV, ROMA score, ADNEX model, and two serum markers (i.e., CA125 and HE4) in both pre-menopausal and post-menopausal women, when applied to all patients presenting with an AM.

The accurate diagnosis of malignancy risk is crucial for ensuring that patients with adnexal masses (AMs) are referred to gynecologic oncology centers for appropriate diagnosis and management, which may include surgery and systemic therapy. Studies have shown that women with ovarian cancer have significantly better outcomes when treated in specialized oncology centers compared to those managed in non-specialist settings [28]. Over the years, several risk prediction models have been developed to more reliably estimate the likelihood of malignancy, which is essential for ensuring that patients receive the most effective and individualized treatment.

In clinical practice, transvaginal ultrasound is commonly employed as the first-line imaging technique for assessing adnexal masses, and most of the well-established risk prediction models are based on ultrasound findings [28,29]. These models play a critical role in guiding patient management by helping clinicians decide if additional diagnostic tests or specialist consultations are necessary and whether surgery should be performed by gynecologic oncologists or can be safely avoided. If an adnexal mass is determined to be benign, it can often be managed with regular ultrasound monitoring rather than surgery. The primary reason to surgically treat benign masses is when they are causing symptoms such as pain or discomfort, and these cases can typically be addressed in local healthcare centers. However, when malignancy is suspected, the patient should be referred to an oncologic referral center for specialized care [4,30].

One of the most effective methods for assessing adnexal masses is the subjective evaluation of pelvic ultrasound images by a highly experienced clinician trained in gynecologic ultrasound. This approach, known as pattern recognition, has been shown to differentiate between benign and malignant pelvic lesions with a high degree of accuracy. In fact, subjective assessment is considered one of the most reliable methods for predicting the likelihood of pelvic malignancy. Unfortunately, this level of expertise is not always readily available in all clinical settings.

Recognizing the need for standardized tools that can be used by clinicians with varying levels of training and expertise, the International Ovarian Tumor Analysis (IOTA) group developed and validated ultrasound-based rules and models to assist in the characterization of adnexal pathology. These models aim to provide reliable support in determining whether a mass is likely benign or malignant, thereby facilitating timely and accurate decision-making for patient care, even in centers without specialized expertise.

In our study population, the RMI I–IV exhibited a lower SE than the ROMA score and the ADNEX model, similar to CA125, and only slightly superior to HE4. However, regardless of menopausal status, RMI I and IV demonstrated high SP and PPV, surpassing all other algorithms. These findings corroborate previous meta-analyses, which consistently reported the high SP of RMI I, exceeding that of ROMA, but also acknowledged its lower SE compared to other models [8,31,32]. The ROMA score showed better SE than RMI I and IV, especially in post-menopausal women, and lower than that of the ADNEX model. The SP of the ROMA model was lower than that RMI I–IV and ADNEX. In relation to the performances (i.e., higher SE than SP) of the ROMA model, our findings align with results from meta-analyses by Dayyani et al. and Suri et al. [33,34]. Moreover, our results are consistent with the meta-analysis by Chacón et al., where ROMA showed better SE but lower SP compared to RMI I [31]. In our study, the ADNEX model demonstrated the highest SE in the diagnosis of malignant AMs (including BOT), irrespective of menopausal status, with a SE of 0.82 and SP of 0.91. The performance of the ADNEX model was higher in the post-menopausal group compared to the pre-menopausal group. Compared to the original and external validation study by Sayasneh et al. [35], in our cohort, the SE of the ADNEX model was slightly lower and the SP was higher.

Recently, the IOTA group compared the diagnostic performance of ADNEX with ROMA [36]. In this study, Landolfo et al. reported AUCs of 0.90 for ADNEX with CA125 and 0.77 for ROMA in premenopausal patients; AUCs were 0.92 for ADNEX with CA125 and 0.89 for ROMA in postmenopausal patients. Our results for SE and SP are comparable, with AUCs for ADNEX with CA125 and ROMA models of 0.90 and 0.76 in premenopausal patients, and 0.87 and 0.93 in postmenopausal patients, respectively. Additionally, our findings align with those of a key study by Meys et al. [8], which compared ADNEX, RMI-I, and subjective assessment (SA). They found that while SA remains superior, ADNEX demonstrated the highest SE, albeit with lower SP. Conversely, RMI had the best SP.

The use of only tumor markers, such as CA125 and HE4, or using the ROMA model may seem like a straightforward and accessible approach to assess AMs without requiring the specialized expertise necessary for TV-US. However, our study results clearly demonstrate that TV-US remains the most accurate method for distinguishing between benign and malignant AMs. This superior diagnostic performance is likely due to the fact that TV-US can capture a broader range of morphological details that tumor markers alone cannot provide.

One significant limitation of using CA125 as a standalone marker is its poor sensitivity in detecting early-stage ovarian cancer. In many cases, CA125 levels may not be elevated in early-stage invasive ovarian malignancies, thus limiting its utility in identifying cancer at its most treatable phase [37,38].

Moreover, conditions unrelated to malignancy, such as endometriosis or pelvic inflammatory disease, can cause elevated CA125 levels, leading to false positives. These conditions often involve inflammation or irritation of the peritoneum, which can trigger an increase in CA125 even in the absence of cancer [39,40]. Similarly, acute or chronic pelvic infections, hemoperitoneum, and even bowel inflammation can all result in CA125 elevations, further complicating the use of this marker in isolation. Despite HE4 being elevated in about 80% of ovarian cancer cases, multiple studies, including meta-analyses, have highlighted its role as a potential complement to CA 125, particularly in distinguishing benign endometriotic and inflammatory lesions in younger women [14,41].

Compared to the literature, our study showed good calibration without significant underestimation or overestimation of malignancy risk for all models. Landolfo et al. reported an underestimation of risk for the ADNEX model (calibration intercept 0.81) and a stronger underestimation for ROMA (calibration intercept 1.20) [36]. However, calibration performance for all validated models varied among different centers, tumor types, and the proportion of malignant cases. Additionally, the ADNEX model showed the highest NB, indicating superior clinical utility across decision thresholds.

### 4.1. Implications for Practice

The ADNEX model holds significant potential to optimize the management of women with adnexal masses (AMs). In fact, this model has demonstrated the highest sensitivity (SE) among available tools, although its specificity (SP) and positive predictive value (PPV) are slightly lower than those of the Risk of Malignancy Index (RMI). This suggests that when ADNEX is used as the first-line approach for differentiating between benign and malignant adnexal masses, there may be a higher incidence of false-positive results [17,28,42]. Therefore, a stepwise strategy aimed at improving SP and PPV to reduce the number of false positives would be highly beneficial for clinical practice.

Three recent studies have explored the application of the ADNEX model as a second-line approach, following Simple Descriptors (SDs) or Simple Rules (SRs), with promising outcomes. Landolfo et al. and the International Ovarian Tumor Analysis (IOTA) group proposed using benign SDs as a first step, followed by the ADNEX model if SDs were not applicable. This two-step strategy yielded a sensitivity of 91% and a specificity of 85.6% when applied at a 10% threshold for ADNEX, suggesting that this combined approach could offer more accurate diagnostic performance. However, the overall diagnostic performance of the two-step strategy was not significantly different from using the ADNEX model alone as the first-line method [43].

Alternatively, two independent trials applied SR as the initial step, followed by ADNEX if SR was inconclusive. Villabona et al. reported a sensitivity of 86.8% and a specificity of 91.01% [44], while Spagnol et al. achieved a sensitivity of 90% and a specificity of 93% [45]. These two-step strategies demonstrated superior diagnostic performance compared to ADNEX alone, achieving similar sensitivity but significantly higher specificity. This improvement effectively reduces the number of false positives, ensuring that fewer patients are unnecessarily referred to oncology units for further evaluation.

In conclusion, the implementation of two-step strategies, incorporating ADNEX alongside other diagnostic tools like SDs or SR, appears to enhance the accuracy of diagnosing adnexal masses. These approaches not only maintain high sensitivity but also improve specificity, thereby refining patient management by reducing false positives and optimizing referrals to oncology units. Adding, Iatrakis et al. introduced a New Risk Malignancy Index (NRMI) for women with pelvic masses, which includes factors like infertility, endometriosis, pregnancy, breastfeeding, ultrasound findings, and the CA125 marker. Larger studies are needed to evaluate its effectiveness due to the small sample size in the initial study [46].

### 4.2. Strengths and Limitations

The main strengths of our study lie in the meticulous data collection process and the strict inclusion criteria. We ensured that only patients who were managed exclusively within our gynecology and oncology departments were included, effectively minimizing biases that could arise from differing diagnostic approaches or surgical techniques across institutions. Additionally, all surgeries were performed within a three-month window following the initial transvaginal ultrasound (TV-US) examination, ensuring consistency in treatment timelines and reducing variability.

Nevertheless, several limitations must be considered. First, the study’s retrospective design could potentially introduce bias. However, to mitigate this, all TV-US predictors were systematically and consecutively documented in our database and electronic hospital records at the point of care, which helps reduce the likelihood of selection bias. A further limitation is the omission of CT scans, as patients with clearly benign AMs only underwent CA 125 testing rather than CT, primarily due to healthcare cost. Another limitation is the fact that our data were collected from a single oncology center. This could limit the generalizability of our findings, as the results may not be directly applicable to other clinical settings with different patient populations or healthcare infrastructures. Despite these limitations, our study provides valuable insights that could inform future research and clinical practice in similar specialized centers.

## 5. Conclusions

In summary, our study represents one of the most comprehensive external validations of the ADNEX model to date, offering a detailed comparison with established diagnostic tools such as the ROMA score, RMI I–IV, and two commonly used serum markers, CA125 and HE4. Our findings demonstrate that the ADNEX model consistently outperforms ROMA, RMI I–IV, CA125, and HE4 in terms of its ability to accurately discriminate between malignant and benign adnexal masses (AMs). Moreover, the ADNEX model offers superior clinical utility, making it a valuable tool for guiding decision-making in the management of patients with suspected ovarian malignancies.

However, while ADNEX excels in overall diagnostic accuracy and clinical applicability, its specificity (SP) remains lower than that of the RMI, indicating a higher rate of false positives. To address this issue, a stepwise diagnostic strategy should be proposed and further investigated.

Further research is warranted to investigate the efficacy of a stepwise strategy, combining ADNEX with other tools to optimize both sensitivity and specificity. 

## Figures and Tables

**Table 1:** Patient and tumor characteristics.

	All	Benign			BOT			Malign		
		All Women	Pre-m Women	Post-m Women	All Women	Pre-m Women	Post-m Women	All Women	Pre-m Women	Post-m Women
Number of patients	581	481	274	207	38	21	17	62	17	45
Age, years ^a^	49 (39–61)	48 (38–60)	40 (32–46)	62 (55–72)	50 (44–58)	44 (34–46)	59 (57–70)	59 (49–71)	45 (41–47)	65 (56–73)
Pre-m women Post-m women	312 (53.7%) 269 (46.3%)	-	-	-	-	-	-	-	-	-
Benign BOT Malignant	481 (82.8%) 38 (6.5%) 62 (10.7%)	-	-	-	-	-	-	-	-	-
FIGO stage: I–II III–IV	-	-	-	-	-	-	-	22 (35.5%) 40 (64.5%)	7 (41.2%) 10 (58.8%)	15 (33.3%) 30 (66.7%)

Legend: Data expressed as *n* (%) or ^a^ median (IQR). BOT: borderline.

**Table 2:** Performance metrics for models and biomarkers at different thresholds.

Biomarker/Model	Threshold	Risk Proportion	Sensitivity (95% CI)	Specificity (95% CI)	PPV	NPV	Youden’s Index	AUC (95% CI)
Biomarker: CA125	0.01	100	1.00 (0.96–1.00)	0.00 (0.00–0.01)	0.17	0.00	0.00	0.76 (0.74–0.79)
Biomarker: CA125	0.03	99.8	1.00 (0.96–1.00)	0.00 (0.00–0.01)	0.17	1.00	0.00	0.76 (0.74–0.79)
Biomarker: CA125	0.05	98.5	0.99 (0.95–1.00)	0.02 (0.01–0.03)	0.17	0.89	0.01	0.76 (0.74–0.79)
Biomarker: CA125	0.10	79.3	0.91 (0.84–0.95)	0.23 (0.20–0.27)	0.20	0.92	0.14	0.76 (0.74–0.79)
Biomarker: CA125	0.15	59.2	0.85 (0.77–0.91)	0.46 (0.42–0.51)	0.25	0.94	0.31	0.76 (0.74–0.79)
Biomarker: CA125	0.20	46.0	0.77 (0.68–0.84)	0.60 (0.56–0.65)	0.29	0.93	0.37	0.76 (0.74–0.79)
Biomarker: CA125	0.25	37.0	0.70 (0.60–0.78)	0.70 (0.66–0.74)	0.33	0.92	0.40	0.76 (0.74–0.79)
Biomarker: CA125	0.30	31.2	0.65 (0.55–0.74)	0.76 (0.72–0.79)	0.36	0.91	0.41	0.76 (0.74–0.79)
Biomarker: CA125	0.40	24.6	0.57 (0.47–0.66)	0.82 (0.78–0.85)	0.40	0.90	0.39	0.76 (0.74–0.79)
Biomarker: CA125	0.50	20.0	0.54 (0.44–0.63)	0.87 (0.84–0.90)	0.47	0.90	0.41	0.76 (0.74–0.79)
Biomarker: HE4	0.01	100	1.00 (0.96–1.00)	0.00 (0.00–0.01)	0.17	0.00	0.00	0.81 (0.78–0.83)
Biomarker: HE4	0.03	100	1.00 (0.96–1.00)	0.00 (0.00–0.01)	0.17	0.00	0.00	0.81 (0.78–0.83)
Biomarker: HE4	0.05	100	1.00 (0.96–1.00)	0.00 (0.00–0.01)	0.17	0.00	0.00	0.81 (0.78–0.83)
Biomarker: HE4	0.10	100	1.00 (0.96–1.00)	0.00 (0.00–0.01)	0.17	0.00	0.00	0.81 (0.78–0.83)
Biomarker: HE4	0.15	98.6	1.00 (0.96–1.00)	0.02 (0.01–0.03)	0.17	1.00	0.02	0.81 (0.78–0.83)
Biomarker: HE4	0.20	97.9	1.00 (0.96–1.00)	0.02 (0.01–0.04)	0.18	1.00	0.02	0.81 (0.78–0.83)
Biomarker: HE4	0.25	94.8	1.00 (0.96–1.00)	0.06 (0.04–0.09)	0.18	1.00	0.06	0.81 (0.78–0.83)
Biomarker: HE4	0.30	89.3	0.98 (0.93–0.99)	0.12 (0.10–0.16)	0.19	0.97	0.10	0.81 (0.78–0.83)
Biomarker: HE4	0.40	60.6	0.89 (0.81–0.94)	0.45 (0.41–0.50)	0.25	0.95	0.34	0.81 (0.78–0.83)
Biomarker: HE4	0.50	36.0	0.74 (0.65–0.82)	0.72 (0.68–0.76)	0.35	0.93	0.46	0.81 (0.78–0.83)
Model: ROMA	0.01	97.1	1.00 (0.96–1.00)	0.04 (0.02–0.06)	0.18	1.00	0.04	0.82 (0.80–0.85)
Model: ROMA	0.03	82.8	0.98 (0.93–0.99)	0.20 (0.17–0.24)	0.20	0.98	0.18	0.82 (0.80–0.85)
Model: ROMA	0.05	64.9	0.87 (0.79–0.92)	0.40 (0.35–0.44)	0.23	0.94	0.27	0.82 (0.80–0.85)
Model: ROMA	0.10	33.0	0.72 (0.63–0.80)	0.75 (0.71–0.79)	0.38	0.93	0.47	0.82 (0.80–0.85)
Model: ROMA	0.15	22.4	0.68 (0.58–0.76)	0.87 (0.84–0.90)	0.52	0.93	0.55	0.82 (0.80–0.85)
Model: ROMA	0.20	17.4	0.63 (0.53–0.72)	0.92 (0.89–0.94)	0.62	0.92	0.55	0.82 (0.80–0.85)
Model: ROMA	0.25	14.6	0.58 (0.48–0.67)	0.94 (0.92–0.96)	0.68	0.92	0.52	0.82 (0.80–0.85)
Model: ROMA	0.30	13.6	0.55 (0.45–0.64)	0.95 (0.93–0.97)	0.70	0.91	0.50	0.82 (0.80–0.85)
Model: ROMA	0.40	9.5	0.42 (0.33–0.52)	0.97 (0.95–0.98)	0.76	0.89	0.39	0.82 (0.80–0.85)
Model: ROMA	0.50	7.6	0.35 (0.26–0.45)	0.98 (0.96–0.99)	0.80	0.88	0.33	0.82 (0.80–0.85)
Model: RMI I	0.01	53.2	0.91 (0.84–0.95)	0.55 (0.50–0.59)	0.29	0.97	0.46	0.86 (0.84–0.88)
Model: RMI I	0.03	53.0	0.91 (0.84–0.95)	0.55 (0.50–0.59)	0.30	0.97	0.46	0.86 (0.84–0.88)
Model: RMI I	0.05	53.0	0.91 (0.84–0.95)	0.55 (0.50–0.59)	0.30	0.97	0.46	0.86 (0.84–0.88)
Model: RMI I	0.10	51.0	0.90 (0.83–0.94)	0.57 (0.53–0.62)	0.30	0.97	0.47	0.86 (0.84–0.88)
Model: RMI I	0.15	47.3	0.88 (0.80–0.93)	0.61 (0.57–0.65)	0.32	0.96	0.49	0.86 (0.84–0.88)
Model: RMI I	0.20	43.0	0.86 (0.78–0.91)	0.66 (0.62–0.70)	0.34	0.96	0.52	0.86 (0.84–0.88)
Model: RMI I	0.25	38.2	0.83 (0.74–0.89)	0.71 (0.67–0.75)	0.37	0.95	0.54	0.86 (0.84–0.88)
Model: RMI I	0.30	35.5	0.80 (0.71–0.87)	0.74 (0.70–0.78)	0.39	0.95	0.54	0.86 (0.84–0.88)
Model: RMI I	0.40	28.6	0.74 (0.65–0.82)	0.81 (0.77–0.84)	0.45	0.94	0.55	0.86 (0.84–0.88)
Model: RMI I	0.50	25.0	0.71 (0.61–0.79)	0.85 (0.81–0.88)	0.49	0.93	0.56	0.86 (0.84–0.88)
Model: RMI IV	0.01	99.7	1.00 (0.96–1.00)	0.00 (0.00–0.02)	0.17	1.00	0.00	0.83 (0.81–0.86)
Model: RMI IV	0.03	99.7	1.00 (0.96–1.00)	0.00 (0.00–0.02)	0.17	1.00	0.00	0.83 (0.81–0.86)
Model: RMI IV	0.05	99.5	1.00 (0.96–1.00)	0.01 (0.00–0.02)	0.17	1.00	0.01	0.83 (0.81–0.86)
Model: RMI IV	0.10	95.0	0.99 (0.95–1.00)	0.06 (0.04–0.08)	0.18	0.97	0.05	0.83 (0.81–0.86)
Model: RMI IV	0.15	88.6	0.98 (0.93–0.99)	0.13 (0.11–0.17)	0.19	0.97	0.11	0.83 (0.81–0.86)
Model: RMI IV	0.20	82.1	0.96 (0.90–0.98)	0.21 (0.17–0.25)	0.20	0.96	0.17	0.83 (0.81–0.86)
Model: RMI IV	0.25	75.9	0.95 (0.89–0.98)	0.28 (0.24–0.32)	0.22	0.96	0.23	0.83 (0.81–0.86)
Model: RMI IV	0.30	69.7	0.94 (0.88–0.97)	0.35 (0.31–0.40)	0.23	0.97	0.29	0.83 (0.81–0.86)
Model: RMI IV	0.40	58.3	0.87 (0.79–0.92)	0.48 (0.43–0.52)	0.26	0.95	0.35	0.83 (0.81–0.86)
Model: RMI IV	0.50	49.1	0.84 (0.76–0.90)	0.58 (0.54–0.63)	0.30	0.95	0.42	0.83 (0.81–0.86)
Model: ADNEX	0.01	94.5	1.00 (0.96–1.00)	0.07 (0.05–0.09)	0.18	1.00	0.07	0.92 (0.90–0.94)
Model: ADNEX	0.03	65.2	0.96 (0.90–0.98)	0.41 (0.37–0.46)	0.25	0.98	0.37	0.92 (0.90–0.94)
Model: ADNEX	0.05	33.6	0.91 (0.84–0.95)	0.78 (0.74–0.82)	0.47	0.98	0.69	0.92 (0.90–0.94)
Model: ADNEX	0.10	21.2	0.82 (0.73–0.88)	0.91 (0.89–0.94)	0.67	0.96	0.74	0.92 (0.90–0.94)
Model: ADNEX	0.15	17.6	0.77 (0.68–0.84)	0.95 (0.92–0.96)	0.76	0.95	0.72	0.92 (0.90–0.94)
Model: ADNEX	0.20	16.0	0.72 (0.63–0.80)	0.96 (0.93–0.97)	0.77	0.94	0.68	0.92 (0.90–0.94)
Model: ADNEX	0.25	14.1	0.63 (0.53–0.72)	0.96 (0.94–0.97)	0.77	0.93	0.59	0.92 (0.90–0.94)
Model: ADNEX	0.30	12.2	0.56 (0.46–0.65)	0.97 (0.95–0.98)	0.79	0.91	0.53	0.92 (0.90–0.94)
Model: ADNEX	0.40	10.7	0.51 (0.41–0.61)	0.98 (0.96–0.99)	0.82	0.91	0.49	0.92 (0.90–0.94)
Model: ADNEX	0.50	9.6	0.47 (0.38–0.57)	0.98 (0.96–0.99)	0.84	0.90	0.45	0.92 (0.90–0.94)

Abbreviations: ADNEX = Assessment of different neoplasia in the adnexa; AUC = area under the receiver operating characteristic (ROC) curve; CA125 = ovarian cancer-related tumor marker; CI = confidence interval; HE4 = human epididymis protein 4; NPV = negative predictive value; PPV = positive predictive value; RMI = risk of malignancy index; ROMA = risk of ovarian malignancy algorithm.

**Table 3:** Performance metrics at recommended cutoffs.

Biomarker/Model	Sensitivity (95% CI)	Specificity (95% CI)	PPV	NPV	AUC (95% CI)
Biomarker: CA125	0.60 (0.54–0.66)	0.80 (0.76–0.83)	0.38	0.91	0.70 (0.67–0.73)
Biomarker: HE4	0.39 (0.30–0.49)	0.96 (0.94–0.98)	0.68	0.88	0.68 (0.65–0.71)
Model: ROMA	0.59 (0.50–0.68)	0.92 (0.88–0.95)	0.60	0.91	0.75 (0.72–0.78)
Model: RMI I	0.56 (0.46–0.65)	0.98 (0.96–0.99)	0.84	0.91	0.77 (0.74–0.80)
Model: RMI IV	0.54 (0.44–0.63)	0.96 (0.94–0.98)	0.76	0.91	0.75 (0.72–0.78)
Model: ADNEX	0.82 (0.73–0.88)	0.91 (0.89–0.94)	0.67	0.96	0.87 (0.85–0.89)

Abbreviations: ADNEX = assessment of different neoplasia in the adnexa; AUC = area under the receiver operating characteristic (ROC) curve; CA125 = ovarian cancer-related tumor marker; CI = confidence interval; HE4 = human epididymis protein 4; NPV = negative predictive value; PPV = positive predictive value; RMI = risk of malignancy index; ROMA = risk of ovarian malignancy algorithm.

## Data Availability

Data are contained within the article and Supplementary Materials.

## Supplementary Materials

The following supporting information can be downloaded at: https://www.mdpi.com/article/10.3390/cancers16223790/s1, Table S1. Pairwise comparisons among biomarkers and models. Table S2. Performance metrics for models and biomarkers at different thresholds in premenopausal patient subset. Table S3. Performance metrics at recommended cutoffs in premenopausal patient subset. Table S4. Performance metrics for models and biomarkers at different thresholds in postmenopausal patient subset. Table S5. Performance metrics at recommended cutoffs in postmenopausal patient subset.
