# Supplementary material for: Clinical Utility and Diagnostic Accuracy of ROMA, RMI, ADNEX, HE4, and CA125 in the Prediction of Malignancy in Adnexal Masses
Source: Cancers (Basel). 2024 Nov 11;16(22):3790. doi: 10.3390/cancers16223790 (PMC11592863; doi:10.3390/cancers16223790)
Supplement: Supplementary file 1 [file cancers-16-03790-s001.zip › cancers-3265123-supplementary.pdf]

SUPPLEMENTARY MATERIAL

INDEX

Table S1. Pairwise comparisons among biomarkers and models.

Table S2. Performance metrics for models and biomarkers at different thresholds in premenopausal patient subset.

Table S3. Performance metrics at recommended cutoffs in premenopausal patient subset.

Table S4. Performance metrics for models and biomarkers at different thresholds in postmenopausal patient subset.

Table S5. Performance metrics at recommended cutoffs in postmenopausal patient subset.

Table S1. Pairwise comparisons among biomarkers and models.

Comparison	AUC difference	CI (lower)	CI (upper)	*p*-value	Correlation
CA125 vs. HE4	-0.043	-0.098	0.012	0.126	0.482
CA125 vs. ROMA	-0.057	-0.108	-0.007	0.026	0.567
CA125 vs. RMI I	-0.096	-0.145	-0.046	0.000	0.536
CA125 vs. RMI IV	-0.068	-0.112	-0.023	0.003	0.656
CA125 vs. ADNEX	-0.156	-0.210	-0.102	0.000	0.380
HE4 vs. ROMA	-0.015	-0.036	-0.007	0.181	0.913
HE4 vs. RMI I	-0.053	-0.104	-0.002	0.043	0.435
HE4 vs. RMI IV	-0.025	-0.075	0.025	0.330	0.515
HE4 vs. ADNEX	-0.113	-0.159	-0.067	0.000	0.472
ROMA vs. RMI I	-0.038	-0.085	0.009	0.112	0.531
ROMA vs. RMI IV	-0.010	-0.052	0.032	0.636	0.660
ROMA vs. ADNEX	-0.098	-0.146	-0.051	0.000	0.459
RMI I vs. RMI IV	0.028	-0.009	0.065	0.144	0.694
RMI I vs. ADNEX	-0.060	-0.095	-0.025	0.001	0.635
RMI IV vs. ADNEX	-0.088	-0.136	-0.040	0.000	0.412

Abbreviations: ADNEX = Assessment of different neoplasias in the adnexa; AUC = Area under the receiver operating characteristic (ROC) curve; CA125 = Ovarian cancer-related tumor marker; CI = Confidence interval; HE4 = Human epididymis protein 4; RMI = Risk of malignancy index; ROMA = risk of ovarian malignancy algorithm.

Overall test *p*-value = 8.77e-09.

Table S2. Performance metrics for models and biomarkers at different thresholds in premenopausal patient subset.

Biomarker/Model	Threshold	Risk proportion	Sensitivity (95% CI)	Specificity (95% CI)	PPV	NPV	Youden's index	AUC (95% CI)	F_1-score
Biomarker: CA125	0.01	1.00	1.00 (0.91 - 1.00)	0.00 (0.00 - 0.01)	0.12	0.00	0.00	0.67 (0.62 - 0.72)	0.22
Biomarker: CA125	0.03	1.00	1.00 (0.91 - 1.00)	0.00 (0.00 - 0.01)	0.12	0.00	0.00	0.67 (0.62 - 0.72)	0.22
Biomarker: CA125	0.05	0.99	1.00 (0.91 - 1.00)	0.01 (0.00 - 0.03)	0.12	1.00	0.01	0.67 (0.62 - 0.72)	0.22
Biomarker: CA125	0.10	0.87	0.92 (0.79 - 0.97)	0.14 (0.10 - 0.18)	0.13	0.93	0.06	0.67 (0.62 - 0.72)	0.23
Biomarker: CA125	0.15	0.71	0.84 (0.70 - 0.93)	0.30 (0.25 - 0.36)	0.14	0.93	0.15	0.67 (0.62 - 0.72)	0.25
Biomarker: CA125	0.20	0.56	0.74 (0.58 - 0.85)	0.47 (0.41 - 0.53)	0.16	0.93	0.20	0.67 (0.62 - 0.72)	0.26
Biomarker: CA125	0.25	0.45	0.66 (0.50 - 0.79)	0.58 (0.52 - 0.64)	0.18	0.92	0.24	0.67 (0.62 - 0.72)	0.28
Biomarker: CA125	0.30	0.36	0.58 (0.42 - 0.72)	0.67 (0.61 - 0.72)	0.20	0.92	0.25	0.67 (0.62 - 0.72)	0.29
Biomarker: CA125	0.40	0.28	0.47 (0.32 - 0.63)	0.75 (0.69 - 0.80)	0.21	0.91	0.22	0.67 (0.62 - 0.72)	0.29
Biomarker: CA125	0.50	0.21	0.45 (0.30 - 0.60)	0.82 (0.78 - 0.87)	0.26	0.91	0.27	0.67 (0.62 - 0.72)	0.33
Biomarker: HE4	0.01	1.00	1.00 (0.91 - 1.00)	0.00 (0.00 - 0.01)	0.12	0.00	0.00	0.77 (0.72 - 0.81)	0.22
Biomarker: HE4	0.03	1.00	1.00 (0.91 - 1.00)	0.00 (0.00 - 0.01)	0.12	0.00	0.00	0.77 (0.72 - 0.81)	0.22
Biomarker: HE4	0.05	1.00	1.00 (0.91 - 1.00)	0.00 (0.00 - 0.01)	0.12	0.00	0.00	0.77 (0.72 - 0.81)	0.22
Biomarker: HE4	0.10	1.00	1.00 (0.91 - 1.00)	0.00 (0.00 - 0.01)	0.12	0.00	0.00	0.77 (0.72 - 0.81)	0.22
Biomarker: HE4	0.15	0.97	1.00 (0.91 - 1.00)	0.03 (0.01 - 0.06)	0.12	1.00	0.03	0.77 (0.72 - 0.81)	0.22
Biomarker: HE4	0.20	0.96	1.00 (0.91 - 1.00)	0.04 (0.03 - 0.07)	0.13	1.00	0.04	0.77 (0.72 - 0.81)	0.22
Biomarker: HE4	0.25	0.92	1.00 (0.91 - 1.00)	0.09 (0.07 - 0.14)	0.13	1.00	0.09	0.77 (0.72 - 0.81)	0.23
Biomarker: HE4	0.30	0.85	0.97 (0.87 - 1.00)	0.17 (0.13 - 0.22)	0.14	0.98	0.15	0.77 (0.72 - 0.81)	0.25
Biomarker: HE4	0.40	0.46	0.76 (0.61 - 0.87)	0.58 (0.52 - 0.63)	0.20	0.95	0.34	0.77 (0.72 - 0.81)	0.32
Biomarker: HE4	0.50	0.21	0.58 (0.42 - 0.72)	0.84 (0.79 - 0.88)	0.33	0.93	0.42	0.77 (0.72 - 0.81)	0.42
Model: ROMA	0.01	0.95	1.00 (0.91 - 1.00)	0.06 (0.04 - 0.10)	0.13	1.00	0.06	0.77 (0.73 - 0.81)	0.23
Model: ROMA	0.03	0.70	0.95 (0.83 - 0.99)	0.33 (0.28 - 0.39)	0.16	0.98	0.28	0.77 (0.73 - 0.81)	0.28
Model: ROMA	0.05	0.44	0.71 (0.55 - 0.83)	0.60 (0.54 - 0.66)	0.20	0.94	0.31	0.77 (0.73 - 0.81)	0.31
Model: ROMA	0.10	0.13	0.42 (0.28 - 0.58)	0.92 (0.88 - 0.94)	0.41	0.92	0.34	0.77 (0.73 - 0.81)	0.42
Model: ROMA	0.15	0.09	0.39 (0.26 - 0.55)	0.95 (0.92 - 0.97)	0.52	0.92	0.34	0.77 (0.73 - 0.81)	0.45
Model: ROMA	0.20	0.07	0.37 (0.23 - 0.53)	0.97 (0.94 - 0.99)	0.64	0.92	0.34	0.77 (0.73 - 0.81)	0.47
Model: ROMA	0.25	0.06	0.34 (0.21 - 0.50)	0.98 (0.96 - 0.99)	0.72	0.91	0.32	0.77 (0.73 - 0.81)	0.46
Model: ROMA	0.30	0.05	0.32 (0.19 - 0.47)	0.98 (0.96 - 0.99)	0.71	0.91	0.30	0.77 (0.73 - 0.81)	0.44
Model: ROMA	0.40	0.04	0.26 (0.15 - 0.42)	0.99 (0.96 - 0.99)	0.71	0.91	0.25	0.77 (0.73 - 0.81)	0.38
Model: ROMA	0.50	0.04	0.24 (0.13 - 0.39)	0.99 (0.96 - 0.99)	0.69	0.90	0.22	0.77 (0.73 - 0.81)	0.35
Model: RMI I	0.01	0.49	0.87 (0.73 - 0.94)	0.56 (0.50 - 0.62)	0.21	0.97	0.43	0.81 (0.77 - 0.85)	0.34
Model: RMI I	0.03	0.49	0.87 (0.73 - 0.94)	0.56 (0.50 - 0.62)	0.22	0.97	0.43	0.81 (0.77 - 0.85)	0.35
Model: RMI I	0.05	0.49	0.87 (0.73 - 0.94)	0.56 (0.50 - 0.62)	0.22	0.97	0.43	0.81 (0.77 - 0.85)	0.35

Model: RMI I	0.10	0.45	0.84 (0.70 - 0.93)	0.60 (0.54 - 0.66)	0.23	0.96	0.44	0.81 (0.77 - 0.85)	0.36
Model: RMI I	0.15	0.39	0.82 (0.67 - 0.91)	0.67 (0.61 - 0.72)	0.25	0.96	0.48	0.81 (0.77 - 0.85)	0.39
Model: RMI I	0.20	0.33	0.76 (0.61 - 0.87)	0.73 (0.67 - 0.78)	0.28	0.96	0.49	0.81 (0.77 - 0.85)	0.41
Model: RMI I	0.25	0.27	0.71 (0.55 - 0.83)	0.79 (0.74 - 0.83)	0.32	0.95	0.50	0.81 (0.77 - 0.85)	0.44
Model: RMI I	0.30	0.24	0.66 (0.50 - 0.79)	0.82 (0.77 - 0.86)	0.33	0.95	0.48	0.81 (0.77 - 0.85)	0.44
Model: RMI I	0.40	0.19	0.58 (0.42 - 0.72)	0.86 (0.82 - 0.90)	0.37	0.94	0.44	0.81 (0.77 - 0.85)	0.45
Model: RMI I	0.50	0.16	0.50 (0.35 - 0.65)	0.89 (0.84 - 0.92)	0.38	0.93	0.39	0.81 (0.77 - 0.85)	0.43
Model: RMI IV	0.01	1.00	1.00 (0.91 - 1.00)	0.00 (0.00 - 0.02)	0.12	1.00	0.00	0.77 (0.72 - 0.81)	0.22
Model: RMI IV	0.03	1.00	1.00 (0.91 - 1.00)	0.00 (0.00 - 0.02)	0.12	1.00	0.00	0.77 (0.72 - 0.81)	0.22
Model: RMI IV	0.05	0.99	1.00 (0.91 - 1.00)	0.01 (0.00 - 0.03)	0.12	1.00	0.01	0.77 (0.72 - 0.81)	0.22
Model: RMI IV	0.10	0.91	0.97 (0.87 - 1.00)	0.10 (0.07 - 0.14)	0.13	0.96	0.07	0.77 (0.72 - 0.81)	0.23
Model: RMI IV	0.15	0.79	0.95 (0.83 - 0.99)	0.23 (0.18 - 0.28)	0.15	0.97	0.18	0.77 (0.72 - 0.81)	0.25
Model: RMI IV	0.20	0.67	0.89 (0.76 - 0.96)	0.36 (0.30 - 0.42)	0.16	0.96	0.25	0.77 (0.72 - 0.81)	0.27
Model: RMI IV	0.25	0.58	0.87 (0.73 - 0.94)	0.46 (0.41 - 0.52)	0.18	0.96	0.33	0.77 (0.72 - 0.81)	0.30
Model: RMI IV	0.30	0.50	0.84 (0.70 - 0.93)	0.55 (0.49 - 0.61)	0.21	0.96	0.39	0.77 (0.72 - 0.81)	0.33
Model: RMI IV	0.40	0.40	0.74 (0.58 - 0.85)	0.65 (0.59 - 0.70)	0.23	0.95	0.39	0.77 (0.72 - 0.81)	0.35
Model: RMI IV	0.50	0.33	0.66 (0.50 - 0.79)	0.72 (0.66 - 0.77)	0.25	0.94	0.38	0.77 (0.72 - 0.81)	0.36
Model: ADNEX	0.01	0.92	1.00 (0.91 - 1.00)	0.09 (0.07 - 0.14)	0.13	1.00	0.09	0.90 (0.87 - 0.94)	0.23
Model: ADNEX	0.03	0.59	0.92 (0.79 - 0.97)	0.46 (0.40 - 0.52)	0.19	0.98	0.38	0.90 (0.87 - 0.94)	0.32
Model: ADNEX	0.05	0.26	0.87 (0.73 - 0.94)	0.83 (0.78 - 0.87)	0.41	0.98	0.70	0.90 (0.87 - 0.94)	0.56
Model: ADNEX	0.10	0.15	0.76 (0.61 - 0.87)	0.94 (0.90 - 0.96)	0.63	0.97	0.70	0.90 (0.87 - 0.94)	0.69
Model: ADNEX	0.15	0.11	0.66 (0.50 - 0.79)	0.97 (0.94 - 0.98)	0.74	0.95	0.63	0.90 (0.87 - 0.94)	0.69
Model: ADNEX	0.20	0.10	0.63 (0.47 - 0.77)	0.97 (0.95 - 0.99)	0.77	0.95	0.61	0.90 (0.87 - 0.94)	0.70
Model: ADNEX	0.25	0.08	0.50 (0.35 - 0.65)	0.98 (0.95 - 0.99)	0.76	0.93	0.48	0.90 (0.87 - 0.94)	0.60
Model: ADNEX	0.30	0.06	0.37 (0.23 - 0.53)	0.99 (0.96 - 0.99)	0.78	0.92	0.35	0.90 (0.87 - 0.94)	0.50
Model: ADNEX	0.40	0.05	0.34 (0.21 - 0.50)	0.99 (0.97 - 1.00)	0.87	0.92	0.33	0.90 (0.87 - 0.94)	0.49
Model: ADNEX	0.50	0.04	0.29 (0.17 - 0.45)	1.00 (0.98 - 1.00)	0.92	0.91	0.29	0.90 (0.87 - 0.94)	0.44

Abbreviations: ADNEX = Assessment of different neoplasias in the adnexa; AUC = Area under the receiver operating characteristic (ROC) curve; CA125 = Ovarian cancer-related tumor marker; CI = Confidence interval; HE4 = Human epididymis protein 4; NPV = Negative predictive value; PPV = Positive predictive value; RMI = Risk of malignancy index; ROMA = risk of ovarian malignancy algorithm.

Table S3. Performance metrics at recommended cutoffs in premenopausal patient subset.

Model	Sensitivity (95% CI)	Specificity (95% CI)	PPV	NPV	Youden's index	AUC (95% CI)	F_1-score
Biomarker: CA125	0.47 (0.40-0.54)	0.72 (0.65-0.79)	0.19	0.91	0.19	0.60 (0.57-0.63)	0.27
Biomarker: HE4	0.39 (0.30-0.49)	0.97 (0.95-0.99)	0.65	0.92	0.37	0.68 (0.65-0.71)	0.49
Model: ROMA	0.42 (0.35-0.50)	0.92 (0.87-0.97)	0.43	0.92	0.34	0.67 (0.64-0.70)	0.43
Model: RMI I	0.37 (0.30-0.45)	1.00 (0.98-1.00)	0.93	0.92	0.37	0.68 (0.63-0.73)	0.53
Model: RMI IV	0.37 (0.29-0.45)	0.98 (0.96-1.00)	0.70	0.92	0.35	0.67 (0.62-0.72)	0.48
Model: ADNEX	0.76 (0.72-0.80)	0.94 (0.92-0.96)	0.63	0.97	0.70	0.85 (0.82-0.88)	0.69

Abbreviations: ADNEX = Assessment of different neoplasias in the adnexa; AUC = Area under the receiver operating characteristic (ROC) curve; CA125 = Ovarian cancer-related tumor marker; CI = Confidence interval; HE4 = Human epididymis protein 4; NPV = Negative predictive value; PPV = Positive predictive value; RMI = Risk of malignancy index; ROMA = risk of ovarian malignancy algorithm.

Table S4. Performance metrics for models and biomarkers at different thresholds in postmenopausal patient subset.

Biomarker/Model	Threshold	Risk proportion	Sensitivity (95% CI)	Specificity (95% CI)	PPV	NPV	Youden's index	AUC (95% CI)	F_1-score
Biomarker: CA125	0.01	1.00	1.00 (0.94 - 1.00)	0.00 (0.00 - 0.02)	0.23	0.00	0.00	0.84 (0.81 - 0.88)	0.37
Biomarker: CA125	0.03	1.00	1.00 (0.94 - 1.00)	0.00 (0.00 - 0.03)	0.23	1.00	0.00	0.84 (0.81 - 0.88)	0.38
Biomarker: CA125	0.05	0.97	0.98 (0.91 - 1.00)	0.03 (0.01 - 0.06)	0.23	0.86	0.01	0.84 (0.81 - 0.88)	0.38
Biomarker: CA125	0.10	0.71	0.90 (0.80 - 0.95)	0.35 (0.29 - 0.42)	0.29	0.92	0.26	0.84 (0.81 - 0.88)	0.44
Biomarker: CA125	0.15	0.45	0.85 (0.75 - 0.92)	0.67 (0.60 - 0.73)	0.44	0.94	0.53	0.84 (0.81 - 0.88)	0.58
Biomarker: CA125	0.20	0.35	0.79 (0.67 - 0.87)	0.79 (0.73 - 0.84)	0.53	0.93	0.58	0.84 (0.81 - 0.88)	0.63
Biomarker: CA125	0.25	0.28	0.73 (0.60 - 0.82)	0.85 (0.80 - 0.89)	0.59	0.91	0.58	0.84 (0.81 - 0.88)	0.65
Biomarker: CA125	0.30	0.26	0.69 (0.57 - 0.79)	0.87 (0.82 - 0.91)	0.62	0.91	0.57	0.84 (0.81 - 0.88)	0.66
Biomarker: CA125	0.40	0.21	0.63 (0.50 - 0.74)	0.92 (0.87 - 0.95)	0.70	0.89	0.55	0.84 (0.81 - 0.88)	0.66
Biomarker: CA125	0.50	0.19	0.60 (0.47 - 0.71)	0.93 (0.89 - 0.96)	0.73	0.89	0.53	0.84 (0.81 - 0.88)	0.65
Biomarker: HE4	0.01	1.00	1.00 (0.94 - 1.00)	0.00 (0.00 - 0.02)	0.23	0.00	0.00	0.82 (0.79 - 0.85)	0.37
Biomarker: HE4	0.03	1.00	1.00 (0.94 - 1.00)	0.00 (0.00 - 0.02)	0.23	0.00	0.00	0.82 (0.79 - 0.85)	0.37
Biomarker: HE4	0.05	1.00	1.00 (0.94 - 1.00)	0.00 (0.00 - 0.02)	0.23	0.00	0.00	0.82 (0.79 - 0.85)	0.37
Biomarker: HE4	0.10	1.00	1.00 (0.94 - 1.00)	0.00 (0.00 - 0.02)	0.23	0.00	0.00	0.82 (0.79 - 0.85)	0.37
Biomarker: HE4	0.15	1.00	1.00 (0.94 - 1.00)	0.00 (0.00 - 0.02)	0.23	0.00	0.00	0.82 (0.79 - 0.85)	0.37
Biomarker: HE4	0.20	1.00	1.00 (0.94 - 1.00)	0.00 (0.00 - 0.02)	0.23	0.00	0.00	0.82 (0.79 - 0.85)	0.37
Biomarker: HE4	0.25	0.99	1.00 (0.94 - 1.00)	0.02 (0.01 - 0.05)	0.23	1.00	0.02	0.82 (0.79 - 0.85)	0.38
Biomarker: HE4	0.30	0.95	0.98 (0.91 - 1.00)	0.06 (0.04 - 0.10)	0.24	0.93	0.05	0.82 (0.79 - 0.85)	0.38
Biomarker: HE4	0.40	0.77	0.97 (0.89 - 0.99)	0.29 (0.23 - 0.36)	0.29	0.97	0.26	0.82 (0.79 - 0.85)	0.45
Biomarker: HE4	0.50	0.53	0.84 (0.73 - 0.91)	0.56 (0.49 - 0.63)	0.36	0.92	0.40	0.82 (0.79 - 0.85)	0.51
Model: ROMA	0.01	1.00	1.00 (0.94 - 1.00)	0.00 (0.00 - 0.02)	0.23	0.00	0.00	0.87 (0.84 - 0.90)	0.37
Model: ROMA	0.03	0.97	1.00 (0.94 - 1.00)	0.03 (0.02 - 0.07)	0.24	1.00	0.03	0.87 (0.84 - 0.90)	0.38
Model: ROMA	0.05	0.90	0.97 (0.89 - 0.99)	0.13 (0.09 - 0.18)	0.25	0.93	0.09	0.87 (0.84 - 0.90)	0.40
Model: ROMA	0.10	0.57	0.90 (0.80 - 0.95)	0.53 (0.46 - 0.60)	0.37	0.95	0.43	0.87 (0.84 - 0.90)	0.52
Model: ROMA	0.15	0.38	0.85 (0.75 - 0.92)	0.77 (0.71 - 0.82)	0.52	0.95	0.62	0.87 (0.84 - 0.90)	0.65
Model: ROMA	0.20	0.29	0.79 (0.67 - 0.87)	0.86 (0.80 - 0.90)	0.62	0.93	0.65	0.87 (0.84 - 0.90)	0.70
Model: ROMA	0.25	0.25	0.73 (0.60 - 0.82)	0.89 (0.84 - 0.93)	0.67	0.92	0.62	0.87 (0.84 - 0.90)	0.70
Model: ROMA	0.30	0.23	0.69 (0.57 - 0.79)	0.91 (0.86 - 0.94)	0.69	0.91	0.60	0.87 (0.84 - 0.90)	0.69
Model: ROMA	0.40	0.15	0.52 (0.39 - 0.64)	0.96 (0.92 - 0.98)	0.78	0.87	0.47	0.87 (0.84 - 0.90)	0.62
Model: ROMA	0.50	0.12	0.42 (0.30 - 0.54)	0.98 (0.94 - 0.99)	0.84	0.85	0.40	0.87 (0.84 - 0.90)	0.56
Model: RMI I	0.01	0.58	0.94 (0.88 - 0.97)	0.53 (0.46 - 0.60)	0.37	0.96	0.47	0.89 (0.86 - 0.91)	0.53
Model: RMI I	0.03	0.58	0.94 (0.85 - 0.97)	0.53 (0.46 - 0.60)	0.37	0.96	0.47	0.89 (0.86 - 0.91)	0.53
Model: RMI I	0.05	0.58	0.94 (0.85 - 0.97)	0.53 (0.46 - 0.60)	0.37	0.96	0.47	0.89 (0.86 - 0.91)	0.53

Model: RMI I	0.10	0.58	0.94 (0.85 - 0.97)	0.53 (0.46 - 0.60)	0.37	0.96	0.47	0.89 (0.86 - 0.91)	0.53
Model: RMI I	0.15	0.57	0.92 (0.82 - 0.97)	0.54 (0.47 - 0.60)	0.37	0.96	0.46	0.89 (0.86 - 0.91)	0.53
Model: RMI I	0.20	0.55	0.92 (0.82 - 0.97)	0.57 (0.50 - 0.63)	0.39	0.96	0.48	0.89 (0.86 - 0.91)	0.55
Model: RMI I	0.25	0.51	0.90 (0.80 - 0.95)	0.61 (0.54 - 0.67)	0.41	0.95	0.51	0.89 (0.86 - 0.91)	0.56
Model: RMI I	0.30	0.49	0.89 (0.78 - 0.94)	0.63 (0.57 - 0.70)	0.42	0.95	0.52	0.89 (0.86 - 0.91)	0.57
Model: RMI I	0.40	0.39	0.84 (0.73 - 0.91)	0.74 (0.68 - 0.79)	0.49	0.94	0.58	0.89 (0.86 - 0.91)	0.62
Model: RMI I	0.50	0.35	0.84 (0.73 - 0.91)	0.79 (0.73 - 0.84)	0.55	0.94	0.63	0.89 (0.86 - 0.91)	0.66
Model: RMI IV	0.01	1.00	1.00 (0.94 - 1.00)	0.00 (0.00 - 0.03)	0.23	1.00	0.00	0.88 (0.85 - 0.91)	0.38
Model: RMI IV	0.03	1.00	1.00 (0.94 - 1.00)	0.00 (0.00 - 0.03)	0.23	1.00	0.00	0.88 (0.85 - 0.91)	0.38
Model: RMI IV	0.05	1.00	1.00 (0.94 - 1.00)	0.00 (0.00 - 0.03)	0.23	1.00	0.00	0.88 (0.85 - 0.91)	0.38
Model: RMI IV	0.10	1.00	1.00 (0.94 - 1.00)	0.00 (0.00 - 0.03)	0.23	1.00	0.00	0.88 (0.85 - 0.91)	0.38
Model: RMI IV	0.15	1.00	1.00 (0.94 - 1.00)	0.00 (0.00 - 0.03)	0.23	1.00	0.00	0.88 (0.85 - 0.91)	0.38
Model: RMI IV	0.20	0.99	1.00 (0.94 - 1.00)	0.01 (0.00 - 0.03)	0.23	1.00	0.01	0.88 (0.85 - 0.91)	0.38
Model: RMI IV	0.25	0.97	1.00 (0.94 - 1.00)	0.04 (0.02 - 0.07)	0.24	1.00	0.04	0.88 (0.85 - 0.91)	0.38
Model: RMI IV	0.30	0.93	1.00 (0.94 - 1.00)	0.10 (0.06 - 0.14)	0.25	1.00	0.10	0.88 (0.85 - 0.91)	0.40
Model: RMI IV	0.40	0.80	0.95 (0.87 - 0.98)	0.25 (0.19 - 0.31)	0.27	0.94	0.20	0.88 (0.85 - 0.91)	0.43
Model: RMI IV	0.50	0.68	0.95 (0.87 - 0.98)	0.40 (0.34 - 0.47)	0.32	0.97	0.35	0.88 (0.85 - 0.91)	0.48
Model: ADNEX	0.01	0.98	1.00 (0.94 - 1.00)	0.03 (0.01 - 0.06)	0.24	1.00	0.03	0.93 (0.91 - 0.95)	0.38
Model: ADNEX	0.03	0.73	0.98 (0.91 - 1.00)	0.35 (0.29 - 0.41)	0.31	0.99	0.33	0.93 (0.91 - 0.95)	0.47
Model: ADNEX	0.05	0.43	0.94 (0.85 - 0.97)	0.72 (0.66 - 0.78)	0.50	0.97	0.66	0.93 (0.91 - 0.95)	0.66
Model: ADNEX	0.10	0.29	0.85 (0.75 - 0.92)	0.88 (0.83 - 0.92)	0.69	0.95	0.74	0.93 (0.91 - 0.95)	0.76
Model: ADNEX	0.15	0.25	0.84 (0.73 - 0.91)	0.92 (0.88 - 0.95)	0.76	0.95	0.76	0.93 (0.91 - 0.95)	0.80
Model: ADNEX	0.20	0.23	0.77 (0.66 - 0.86)	0.93 (0.89 - 0.96)	0.77	0.93	0.71	0.93 (0.91 - 0.95)	0.77
Model: ADNEX	0.25	0.21	0.71 (0.59 - 0.81)	0.94 (0.90 - 0.96)	0.77	0.92	0.65	0.93 (0.91 - 0.95)	0.74
Model: ADNEX	0.30	0.20	0.68 (0.55 - 0.78)	0.95 (0.91 - 0.97)	0.79	0.91	0.62	0.93 (0.91 - 0.95)	0.73
Model: ADNEX	0.40	0.17	0.61 (0.49 - 0.72)	0.96 (0.92 - 0.98)	0.81	0.89	0.57	0.93 (0.91 - 0.95)	0.70
Model: ADNEX	0.50	0.16	0.58 (0.46 - 0.70)	0.96 (0.93 - 0.98)	0.82	0.88	0.54	0.93 (0.91 - 0.95)	0.68

Abbreviations: ADNEX = Assessment of different neoplasias in the adnexa; AUC = Area under the receiver operating characteristic (ROC) curve; CA125 = Ovarian cancer-related tumor marker; CI = Confidence interval; HE4 = Human epididymis protein 4; NPV = Negative predictive value; PPV = Positive predictive value; RMI = Risk of malignancy index; ROMA = risk of ovarian malignancy algorithm.

Table S5. Performance metrics at recommended cutoffs in postmenopausal patient subset.

Biomarker/Model	Sensitivity (95% CI)	Specificity (95% CI)	PPV	NPV	Youden's index	AUC (95% CI)	F_1-score
Biomarker: CA125	0.68 (0.64-0.72)	0.91 (0.87-0.94)	0.69	0.90	0.59	0.79 (0.75-0.83)	0.68
Biomarker: HE4	0.39 (0.30-0.48)	0.95 (0.93-0.98)	0.71	0.84	0.34	0.67 (0.63-0.70)	0.50
Model: ROMA	0.69 (0.63-0.75)	0.91 (0.88-0.94)	0.69	0.91	0.60	0.80 (0.77-0.83)	0.69
Model: RMI I	0.68 (0.64-0.72)	0.95 (0.93-0.97)	0.81	0.91	0.63	0.82 (0.79-0.85)	0.74
Model: RMI IV	0.65 (0.61-0.69)	0.95 (0.92-0.97)	0.78	0.90	0.59	0.80 (0.77-0.83)	0.71
Model: ADNEX	0.85 (0.83-0.88)	0.88 (0.85-0.91)	0.69	0.95	0.74	0.87 (0.84-0.90)	0.76

Abbreviations: ADNEX = Assessment of different neoplasias in the adnexa; AUC = Area under the receiver operating characteristic (ROC) curve; CA125 = Ovarian cancer-related tumor marker; CI = Confidence interval; HE4 = Human epididymis protein 4; NPV = Negative predictive value; PPV = Positive predictive value; RMI = Risk of malignancy index; ROMA = risk of ovarian malignancy algorithm.
